# The Health of the Nation: The Essential Food to Maintain Health

**Published:** 1919-02-08

**Authors:** Thomas G. Read

**Affiliations:** Hillside, Blackgang, Isle of Wight.


*To the Editor of* THE HOSPITAL.

### The Health of the Nation: The Essential Food to Maintain Health.

*To the Editor of* THE HOSPITAL.

SIR,—Formerly we were a healthy and contented race. Now we are becoming a race predisposed to disease and restlessness. Medical examination has during the war shown like a searchlight an appalling amount of physical incapacity in our people. The cause is not difficult to discover. You cannot rear healthy animals and birds unless they have suitable food. For nearly fifty years much of our food has been becoming less nutritious than formerly. All foods in their natural state contain digestive enzymes. These, under the influence of moderate warmth and suitable moisture, render the nutritive parts of food soluble by causing them to take up water and undergo deduplication that they may pass through the walls of the blood capillaries, be assimilated, and nourish the body. It is the quantity of food assimilated that promotes nutrition, not the quantity taken.

In roller milling the wheat germs containing the enzymes amylase and cellulose are removed from the flour during milling. With the old stone mill the unimpaired enzymes of the grain were retained in the flour. Owing to the actions of the food, enzymes having been to a large extent disregarded since the introduction of the roller mill into the United Kingdom in 1862, the enzymes of the digestive tract have been expected to act upon more nutritive material than they are able to, with the result much undigested cellulose, fat, proteid, and starch have been evacuated from the bowels, which would have been assimilated to nourish the body if the food enzymes had acted. Moreover, when non-enzymic carbohydrate food is in the mouth, and while passing down the œsophagus, acid is formed which combines with alkaline bases contained in the food, forming neutral salts, which are chiefly excreted by the kidneys and the skin, and

**The Editor's Letter-Box**—(*continued*).

the organism deprived of these essential substances. It is obvious alkaline bases prescribed by the medical practitioner will also be acted upon by the acid. Some of the acid formed during eating decays the teeth by combining with the lime salts they contain.

In 1917, Mr. Arnold F. Hills, the President of the Vegetarian Federal Union, approached eighty Fellows of the Royal Society for information concerning the effects of food enzymes upon nutrition. None of these was able to furnish him with the information he asked for. Apparently none of these scientists had studied the effects of the actions of food enzymes. The enzymes of the digestive tract alone cannot maintain complete nutrition. This has been demonstrated by the writer by experiments on animals and birds. For example, with puppies and kittens the health became impaired when they were fed on bread and milk made with boiled milk and bread produced from flour not containing the enzymes of the grain. They also did badly when given new milk to which preservatives had been added. Preservatives destroy the enzymes. Dogs, when fed solely on frozen meat, died from dysentery. Extreme cold destroys the enzymes. The enzymes are destroyed when food is kept in cold storage. Pigs did badly when they were sty-fed with boiled blighted potatoes. Blight destroys the enzymes. When the potatoes were mashed and when nearly cold mixed with meal containing the unimpaired enzymes of the grain, the pigs recovered. The enzymes acted upon the nutritive constituents of the blighted potatoes. Young chickens, when fed on meal baked to destroy the enzymes, soon all died. Laying hens soon stopped laying when fed with baked grain.

In the above-mentioned experiments no substance was removed from any of the foods, only the enzymic activity was destroyed, and then in all cases the food was found to be harmful to health. The nation is now being supplied with a very great amount of non-enzymic food, such as bread made from flour not containing the enzymes owing to the grains used having been baked to destroy the enzymic activity before milling; oatmeal produced from kiln-dried oats; frozen meat; and non-enzymic margarine, instead of butter containing an important enzymic fat soluble, which greatly promotes growth and well-being, and particularly healthy reproduction. It has recently been noticed that children are checked in their growth when given margarine instead of butter. No wonder medical examination during the past four years has demonstrated an excessive amount of impaired vitality in our people.—I am, Sir, faithfully yours,

Hillside, Blackgang, THOMAS G. READ.

Isle of Wight. January 30, 1919.

